# The Two-Stage Suspension System of the Fiber Optic Vector Hydrophone for Isolating the Vibration from the Mooring Rope

**DOI:** 10.3390/s22239261

**Published:** 2022-11-28

**Authors:** Yichi Zhang, Zhou Meng, Jianfei Wang, Mo Chen, Yan Liang, Xiaoyang Hu

**Affiliations:** 1 College of Meteorology and Oceanology, National University of Defense Technology, Changsha 410073, China; 2 Academy of Artillery and Air Defense, Nanjing 210000, China

**Keywords:** two-stage suspension system, fiber optic vector hydrophone, acceleration transmissibility, acceleration response

## Abstract

The two-stage suspension system (TSSS) is designed for the fiber optic vector hydrophone (FOVH) to isolate the vibration from the mooring rope. The acceleration transmissibility of the TSSS is studied theoretically and experimentally. The results show that the TSSS has a major advantage over the traditional one-stage suspension system (OSSS). Typically, the vibration isolation of the TSSS is demonstrated to be over 25 dB higher than that of the OSSS at 100 Hz. Meanwhile, it is demonstrated that the TSSS has little negative influence on the in-band acceleration response of the FOVH. The TSSS has the prospect of reducing the mechanical noise of the FOVH, which is conducive to suppressing the self-noise and enhancing the ability of weak signal detection.

## 1. Introduction

A fiber optic sensor has the unique advantages of small size, high sensitivity, light weight, being immune to electromagnetic interference, and being easy to multiplex. Therefore, it has been widely used to measure various physical parameters of interest, such as pressure [1,2,3,4,5], temperature [4,5,6,7,8,9], salinity [7,10,11], as well as some kinematic parameters [12,13,14,15,16]. The fiber optic accelerometer, which takes the acceleration as the measurand, is one type of fiber optic sensor. Since first reported in the early 1980s [17], it has undergone a fast development. At present, many types of fiber optic accelerometers have been reported, which can be sorted into the interferometric type [14,15,16,18], Bragg-grating type [19,20,21], and fiber-laser type [22,23,24] in terms of the sensing mechanism. Different types have different advantages. Generally, the Bragg-grating type and fiber-laser type are convenient for miniaturization and perform better in low-frequency detection. The interferometric type is earlier to be developed and more mature, which has the merits of high sensitivity, wide band, and simple structure.

In general, the applications of the fiber optic accelerometers include the seismometer [25,26,27] and hydrophone [18,28]. In the field of hydrophone, the fiber optic accelerometer is usually referred to as the fiber optic vector hydrophone (FOVH), which takes the vector information of the underwater sound field as the measurand [18,29]. Different from the conventional piezoelectric-type vector hydrophone, the FOVH shares the same merits with the fiber optic sensors, while it has some unique advantages over the fiber optic scalar hydrophone, such as the enhancement of the gain, intrinsic dipole directivity [30], and being free from the “blurred starboard side” [28], etc. Therefore, the FOVH is well suitable for the target azimuth estimation in the underwater detection.

As for the co-vibrating-type of FOVH, the optimal density of the hydrophone is required to be equal to the that of the surrounding water, that is, zero buoyancy. However, the actual density of the ambient water is usually flexible, making the zero-buoyancy condition not easy to meet, especially in the sea application. Therefore, it is necessary to suspend the vector hydrophone onto the mooring rope of the array for a position-fixed vibration [31]. In general, the suspension is flexible to avoid interfering with the signal pick-up as much as possible, which is also conducive to suppressing the vibration from the attached mooring rope. The simplest structure of the flexible suspension system is a one-stage suspension system (OSSS), in which the vector hydrophone is hung on the frame directly with several springs. The adoption of the OSSS to the vector hydrophone can be traced back to the 1950s [32]. A similar OSSS has been adopted for over half a century. In 2003, a detailed theoretical analysis on the OSSS of the vector hydrophone was reported [33]. In 2006, Abraham [34] and Clark [35] reported the other type of the OSSS in their vertical line array with five vector hydrophones, where they used urethane hoses as the suspension devices instead of springs. In addition, also in 2006, McEachern reported a deep ocean vector sensor research array, which adopted OSSS as well. The reported suspension devices were springs [36]. The above reports on the suspension system are almost all within the field of the piezoelectric-type vector hydrophone because the piezoelectric-type vector hydrophone is the earliest to be used in underwater detection. As for the FOVH, the suspension system in the practical application has not been reported to the best of our knowledge, but Jin [37] and Wang [38] measured the acceleration sensitivities of their designed FOVHs in the laboratory tests, where they used the OSSS to suspend the FOVHs under test.

For a well-designed suspension system, it should have the ability to isolate the vibration from the attached mooring rope, so as to suppress the mechanical noise [31]. Although traditional OSSS can meet the demand of many applications, its ability of mechanical noise suppression is not enough when faced with the increasing demand of the weak signal detection [39,40,41]. A suspension system with a lower vibration transmissibility is in demand.

In this paper, a two-stage suspension system (TSSS) is designed for an interferometric acceleration-type FOVH to reduce the vibration transmissibility from the mooring rope, which has not been reported to the best of our knowledge. The acceleration transmissibility of the TSSS is studied theoretically and experimentally. The results show that it performs significantly better in isolating the vibration from the mooring rope and the outer frame. Therefore, TSSS has a potential prospect in suppressing the mechanical noise of the FOVH, which is conducive to enhancing the ability of weak signal detection.

## 2. Theory

As for the acceleration-type co-vibrating FOVH, the acceleration transmissibility T reflects the performance of the vibration isolation. It is the ratio of the acceleration achieved by the FOVH to the acceleration on the outer frame, and the equation of definition is

(1) $$T\left(\omega \right)=\frac{a_{A}}{a_{F}},$$

in which $a_{A}$ is the acceleration achieved by the FOVH, and $a_{F}$ is the acceleration on the outer frame. It should be noted that the outer frame of the FOVH is considered to be fixed on the mooring rope firmly, so the acceleration from the mooring rope is ideally equal to the acceleration on the outer frame in the theoretical analysis.

Meanwhile, the parameter $T_{F}$ that indicates the acceleration response of the FOVH is also defined, which is

(2) $$T_{F}\left(\omega \right)=\frac{ma_{P}}{F_{0}},$$

in which m is the mass of the FOVH, $F_{0}$ is the amplitude of the ambient exciting force, and $a_{P}$ is the acceleration picked by the FOVH under the exciting force $F_{0}$. Ideally, $T_{F}\left(\omega \right)$ should be equal to 1, but the suspension system makes it deviate from the value, especially when the frequency of interest is near the resonant frequency of the suspension system.

### 2.1. The Equivalence of the Suspension System Composed of Several Springs with Difference Suspending Angles

The equivalence of the suspension system, which is composed of several springs with different suspending angles, is key for the theoretical analysis. The method of vector synthesis is not applicable in the equivalence. Instead, the energy conservation method should be used [42].

**Figure 1:**
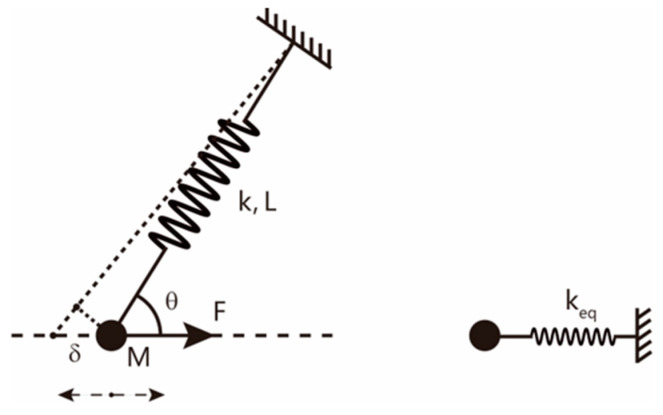
The schematic of one example spring oscillator system that is used to achieve the equivalence of the stiffness.

The spring oscillator system shown in Figure 1 is taken as the example for the equivalence process. The system is composed of the spring with the stiffness of k and the sphere with the mass of *M*. Neglecting the gravity, the dynamic force F drives mass *M* to vibrate along the direction of F. The angle between the force F and the suspension spring is θ. Assume that the small displacement of the mass *M* from the equilibrium position along the direction of F is δ, the corresponding displacement along the suspension direction is δcosθ. Under such circumstance, the stored elastic potential energy due to the displacement of the mass *M* is $k{\left(\delta \cos\theta \right)}^{2}/2$, which should be equal to the energy stored by the equivalent spring oscillator system. Then, the equivalent equation is

(3) $$\frac{1}{2}k{\left(\delta \cos\theta \right)}^{2}=\frac{1}{2}k_{eq}\delta^{2}.$$

Therefore, the equivalent stiffness along the direction of F is

(4) $$k_{eq}=k\cos^{2}\theta .$$

When more springs with various suspension angles and stiffnesses are attached to the sphere, the equivalent equation is extended to be

(5) $$\overset{n}{\underset{i=1}{\sum }}\left[\frac{1}{2}k_{i}{\left(\delta \cos\theta_{i}\right)}^{2}\right]=\frac{1}{2}k_{eq}\delta^{2}.$$

Then, we can get the general expression of the equivalent stiffness, which is

(6) $$k_{eq}=\overset{n}{\underset{i=1}{\sum }}k_{i}\cos^{2}\theta_{i}.$$

It is observed in Equation (6) that the equivalent stiffness along the direction of F is mainly determined by the suspending angles and the stiffnesses of the practical springs.

**Figure 2:**
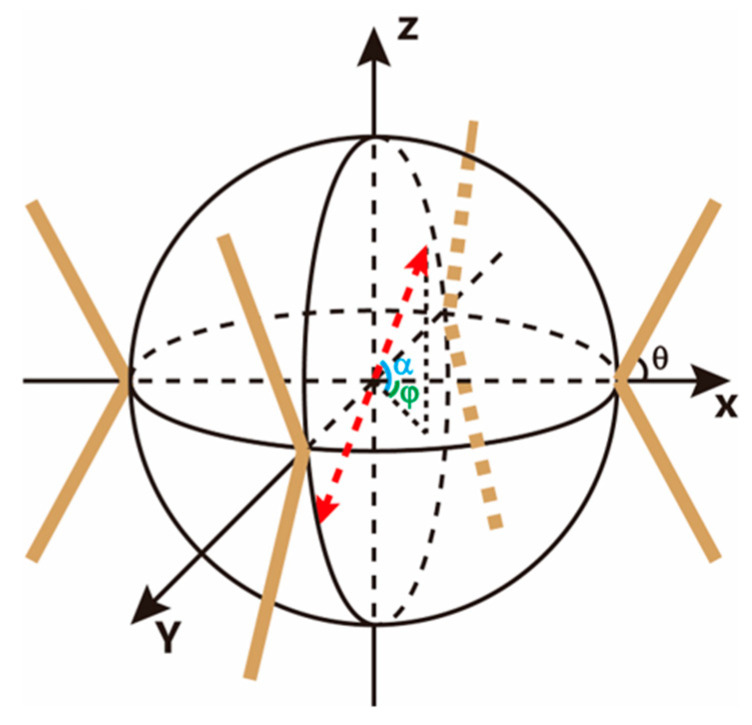
The schematic of one-stage suspended FOVH system. The angle *θ* is the suspending angle, *α* is the polar angle of the excitation, and *φ* is the azimuth angle of the excitation.

One type of the OSSS for the FOVH is shown in Figure 2, which is the schematic diagram of the OSSS used in the subsequent research. In the system, eight springs with the same stiffnesses k and damping coefficients c suspend the FOVH symmetrically. The suspending angles are all θ. In the spherical coordinates established by the sensing principal axes of the FOVH, eight suspending directions can be described by eight unit vectors, which are (1,θ,0), (1,π−θ,0), (1,θ,π/2), (1,π−θ,π/2), (1,θ,π), (1,π−θ,π), (1,θ,3π/2), and (1,π−θ,3π/2), respectively. They correspond to the vectors in the Cartesian coordinates as (sinθ,0,cosθ), (0,sinθ,cosθ), (−sinθ,0,cosθ), (0,−sinθ,cosθ), (sinθ,0,−cosθ), (0,sinθ,−cosθ), (−sinθ,0,−cosθ), (0,−sinθ,−cosθ). Assume that the exciting direction is expressed by the unit vector (1,α,φ), it corresponds to the vector  in the Cartesian coordinates, where the angle α is the polar angle and φ is the azimuth angle. Then, we can easily get the equivalent stiffness, which is

(7) $$k_{eq}=\left(4\sin^{2}\theta \;\sin^{2}\alpha +8\cos^{2}\theta \;\cos^{2}\alpha \right)k.$$

Apparently, when the suspending angle is fixed, the equivalent stiffness is only associated with the polar angle α of the exciting direction, and has no relationship with the azimuth angle φ. This can be attributed to the fact that the suspension system is symmetric in the horizontal plane. From Equation (7), we can get the equivalent stiffnesses along the vertical direction and any horizontal directions, which are

(8) $$k_{eq}^{⊥}=8k\;\cos^{2}\theta ,\;k_{eq}^{∥}=4k\;\sin^{2}\theta .$$

Likewise, the equivalent damping coefficient is

(9) $$c_{eq}=\left(4\sin^{2}\theta \;\sin^{2}\alpha +8\cos^{2}\theta \;\cos^{2}\alpha \right)c,$$

and the equivalent damping coefficients along the vertical direction and any horizonal directions are

(10) $$c_{eq}^{⊥}=8c\;\cos^{2}\theta ,\;c_{eq}^{∥}=4c\;\sin^{2}\theta .$$

### 2.2. The Theoretical Model of the OSSS

**Figure 3:**
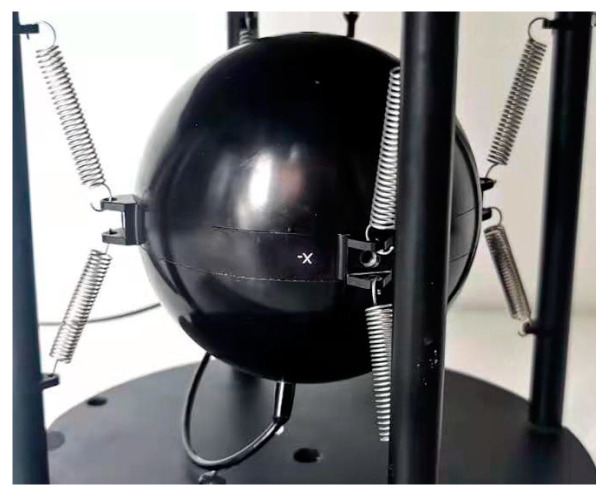
The structural image of the OSSS assembly.

The image of the one-stage suspended FOVH used in the paper is shown in Figure 3. In the structure, the FOVH is simply hung on the outer frame with eight identical springs. The suspending angles are the same for all the springs.

#### 2.2.1. The Acceleration Transmissibility Model of the OSSS

**Figure 4:**
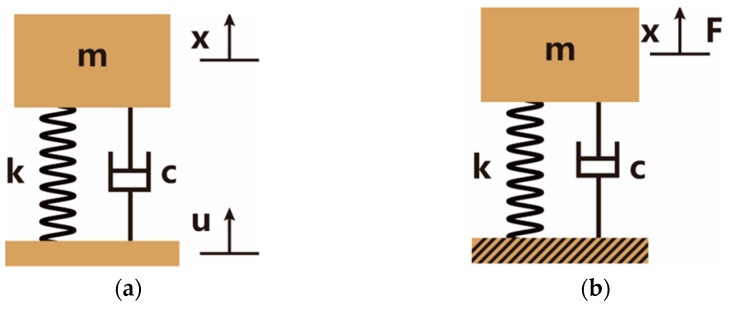
The equivalent model of the OSSS: (**a**) the acceleration transmissibility model; (**b**) the acceleration response model. The parameters k and c are the equivalent stiffness and damping coefficient along the exciting direction, m is the mass of the FOVH, *x* is the displacement of the FOVH, u is the displacement of the outer frame in the acceleration transmissibility model, and F is the ambient exciting force provided by the signal in the acceleration response model.

Through the equivalence, the acceleration transmissibility model of the OSSS can be simplified as the single-degree-of-freedom spring oscillator. The equivalent model is illustrated in Figure 4a. In the model, the parameters k and c are the equivalent stiffness and damping coefficient along the exciting direction, m is the mass of the FOVH, x is the displacement of the FOVH, and u is the displacement of the outer frame.

In the equivalent model, the motion of the FOVH is controlled by the motion equation [42]

(11) $$m\ddot{x}+c\dot{x}+kx=c\dot{u}+ku.$$

Assume that the harmonic motion of the outer frame is $u=Ue^{j\omega t}$, then the motion of the FOVH in the steady state case is also harmonic, which is assumed to be $x=Xe^{j\omega t}$. In the expressions, j is the imaginary unit, ω is the circular frequency of the excitation, while U and X are all the complex amplitudes. Take them into Equation (11) and we have

(12) $$-m\omega^{2}X+j\omega cX+kX=j\omega cU+kU.$$

Then, we can get the acceleration transmissibility expressed as

(13) $$\left|T\right|=\sqrt{\frac{1+{\left(2\zeta \eta \right)}^{2}}{{\left(1-\eta^{2}\right)}^{2}+{\left(2\zeta \eta \right)}^{2}}},$$

where $\zeta =c/2\sqrt{mk}$ is the damping ratio, $\eta =\omega /\omega_{0}$ is the frequency ratio, and $\omega_{0}=\sqrt{k/m}$ is the undamped natural frequency.

#### 2.2.2. The Acceleration Response Model of FOVH in the OSSS

When considering the acceleration response of the FOVH suspended in the OSSS, the outer frame can be regarded as a fixed foundation because the signal is relatively weak. The equivalent model is shown in Figure 4b. In the model, the parameters k and c are the equivalent stiffness and damping coefficient along the exciting direction, m is the mass of the FOVH, x is the displacement of the FOVH, and F is the ambient exciting force provided by the signal.

In the equivalent model, the motion of the FOVH is controlled by the motion equation [42]

(14) $$m\ddot{x}+c\dot{x}+kx=F_{0}e^{j\omega t}$$

where $F_{0}$ and ω are the amplitude and circular frequency of the harmonic exciting force. From Equation (14), we can easily get the acceleration response of the FOVH, which is

(15) $$\left|T_{F}\right|=\frac{\eta^{2}}{\sqrt{{\left(1-\eta^{2}\right)}^{2}+{\left(2\zeta \eta \right)}^{2}}}$$

### 2.3. The Theoretical Model of the TSSS

**Figure 5:**
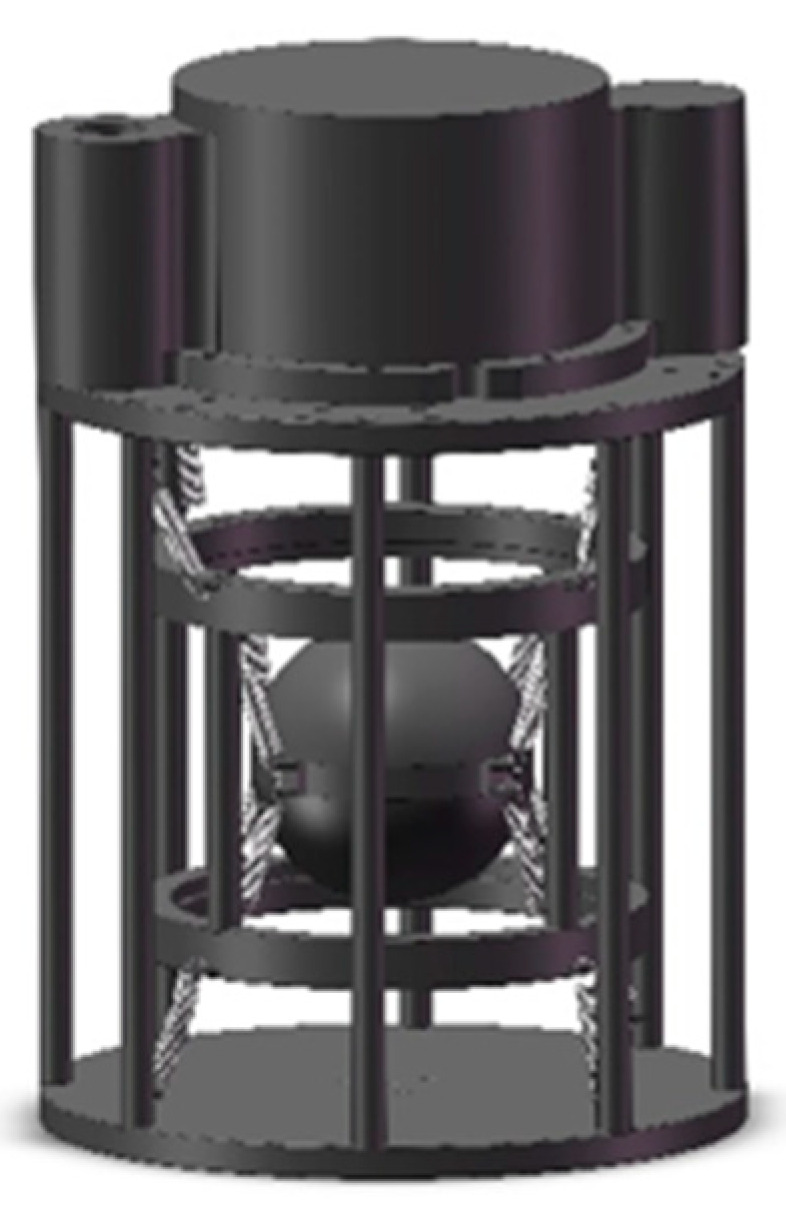
The schematic diagram of the TSSS assembly.

The structure of the TSSS for the FOVH is shown in Figure 5. We add a hollow-cylindrical-type middle frame between the outer frame and the FOVH to form the TSSS. The FOVH is suspended on the middle frame with eight identical springs, while the middle frame is suspended on the outer frame in the same way. The suspending angles are all the same as those in the OSSS. For convenience, we define the suspension between the middle frame and the FOVH as the inner suspension, and that between the middle frame and outer frame as the outer suspension.

#### 2.3.1. The Acceleration Transmissibility Model of the TSSS

**Figure 6:**
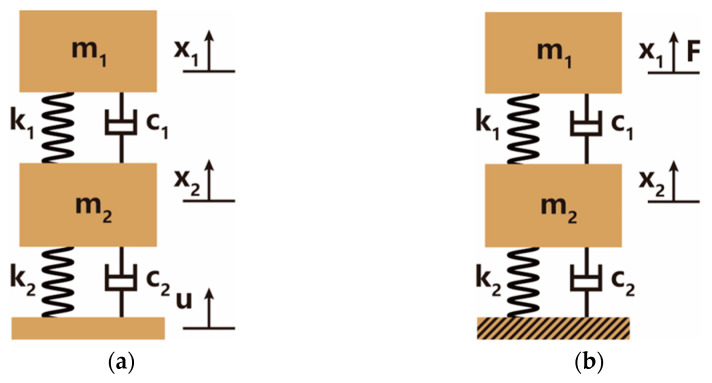
The equivalent model of the TSSS: (**a**) the acceleration transmissibility model; (**b**) the acceleration response model. The parameters $k_{1}$ and $c_{1}$ are the equivalent stiffness and damping coefficient of the inner suspension along the exciting direction, while $k_{2}$ and $c_{2}$ are those of the outer suspension. Other parameters are defined as follows: $m_{1}$ and $x_{1}$ are the mass and displacement of the FOVH, $m_{2}$ and $x_{2}$ are the mass and displacement of the middle frame, u is the displacement of the outer frame. In the acceleration transmissibility model, F is the ambient exciting force provided by the signal in the acceleration response model.

The acceleration transmissibility model of the TSSS is the dual-degree-of-freedom spring oscillator after the equivalence, which is illustrated in Figure 6a. In the model, the parameters $k_{1}$ and $c_{1}$ are the equivalent stiffness and damping coefficient of the inner suspension along the exciting direction, while $k_{2}$ and $c_{2}$ are those of the outer suspension. Other parameters are defined as follows: $m_{1}$ and $x_{1}$ are the mass and displacement of the FOVH, $m_{2}$ and $x_{2}$ are the mass and displacement of the middle frame, and u is the displacement of the outer frame.

In the equivalent model, the motion of the FOVH in the TSSS is controlled by the equation set expressed as

(16) $$\{\begin{array}{l} m_{1}\ddot{x_{1}}+c_{1}\dot{x_{1}}+k_{1}x_{1}-c_{1}\dot{x_{2}}-k_{1}x_{2}=0\\ m_{2}\ddot{x_{2}}+\left(c_{1}+c_{2}\right)\dot{x_{2}}+\left(k_{1}+k_{2}\right)x_{2}-c_{1}\dot{x_{1}}-k_{1}x_{1}=c_{2}\dot{u}+k_{2}u \end{array}$$

Despite that the equation is significantly more complicated, we can solve it with the method described in the OSSS model as well. For convenience, we assume that the equivalent stiffness and damping coefficient are the same for the inner frame and outer frame, which are k and c, respectively. Under this circumstance, the acceleration transmissibility acquired from Equation (16) is

(17) $$\left|T\right|=\frac{1+{\left(2\zeta_{1}\eta_{1}\right)}^{2}}{\sqrt{{\left[1-{\left(2\zeta_{1}\eta_{1}\right)}^{2}+\eta_{1}^{4}\chi -\left(2+\chi \right)\eta_{1}^{2}\right]}^{2}+{\left[4-2\chi \eta_{1}^{2}-4\eta_{1}^{2}\zeta_{1}\eta_{1}\right]}^{2}}},$$

where $\zeta_{1}=c/2\sqrt{m_{1}k}$ is the damping ratio of the inner suspension, $\eta_{1}=\omega /\omega_{1}$ is the frequency ratio of the inner suspension, $\omega_{1}=\sqrt{k/m_{1}}$ is the undamped natural frequency of the inner suspension, and $\chi =m_{2}/m_{1}$ is the mass ratio of the middle frame to the FOVH. From Equation (17), we can find that the acceleration transmissibility of the TSSS is related to not only the damping ratio $\zeta_{1}$ and undamped natural frequency $\omega_{1}$ but also the mass ratio χ.

#### 2.3.2. The Acceleration Response Model of FOVH in the TSSS

The same as the one-stage acceleration response model, the outer frame can also be regarded as a fixed foundation in the two-stage acceleration response model. The equivalent model is shown in Figure 6b, which is also a dual-degree-of-freedom spring oscillator. In the model, the parameters $k_{1}$ and $c_{1}$ are the equivalent stiffness and damping coefficient of the inner suspension along the exciting direction, while $k_{2}$ and $c_{2}$ are those of the outer suspension. Other parameters are defined as follows: $m_{1}$ and $x_{1}$ are the mass and displacement of the FOVH, $m_{2}$ and $x_{2}$ are the mass and displacement of the middle frame, and *F* is the ambient exciting force provided by the signal.

In the equivalent model, the motion of the FOVH is controlled by the motion equation set expressed as

(18) $$\{\begin{array}{l} m_{1}\ddot{x_{1}}+c_{1}\dot{x_{1}}+k_{1}x_{1}-c_{1}\dot{x_{2}}-k_{1}x_{2}=F_{0}e^{j\omega t}\\ m_{2}\ddot{x_{2}}+\left(c_{1}+c_{2}\right)\dot{x_{2}}+\left(k_{1}+k_{2}\right)x_{2}-c_{1}\dot{x_{1}}-k_{1}x_{1}=0 \end{array}.$$

Assume that the equivalent stiffness and damping coefficient of the inner suspension is the same as those of the outer suspension, which are k and c. Then, we can achieve the acceleration response of the FOVH by solving Equation (18), which is

(19) $$\left|T_{F}\right|=\eta_{1}^{2}\sqrt{\frac{{\left(2-\chi \eta_{1}^{2}\right)}^{2}+{\left(4\zeta_{1}\eta_{1}\right)}^{2}}{{\left[1-\left(\chi +2\right)\eta_{1}^{2}+\chi \eta_{1}^{4}-{\left(2\zeta_{1}\eta_{1}\right)}^{2}\right]}^{2}+{\left[4\zeta_{1}\eta_{1}-2\left(\chi +2\right)\zeta_{1}\eta_{1}^{3}\right]}^{2}}}.$$

Likewise, the acceleration response is also related to the damping ratio $\zeta_{1}$, undamped natural frequency $\omega_{1}$, and the mass ratio χ.

## 3. Simulation and Analysis

### 3.1. Simulation According to the Theory

#### 3.1.1. Comparison of the Acceleration Transmissibilities of the Two Suspension Systems

(a) The mass ratio is fixed, and the damping ratio is changed.

**Figure 7:**
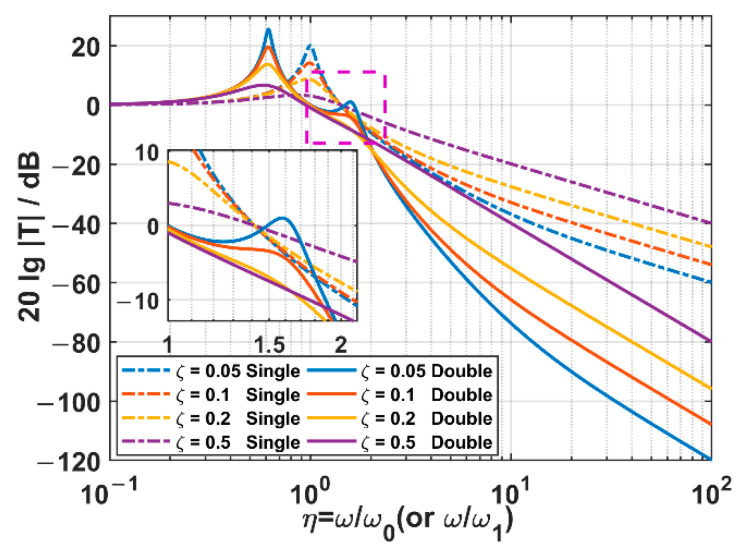
The acceleration transmissibility spectra of two suspension systems in different damping ratio cases. Among them, the dashed lines are the results of the OSSS, while the solid lines are those of the TSSS.

In this part, the mass ratio χ is fixed as 1, and the damping ratio ζ (or $\zeta_{1}$ in the TSSS case) is set as 0.05, 0.1, 0.2, and 0.5, respectively. The acceleration transmissibility spectra of two kinds of suspension systems are sketched according to Equations (13) and (17), which are illustrated in Figure 7.

As for the OSSS, there are those significant characteristics as follows:

1. When the frequency ratio $\eta \leq \sqrt{2}$, the system works in the resonant region, and it is not able to suppress the acceleration transmitted from the outer frame;
2. When the frequency ratio $\eta >\sqrt{2}$, the system turns into a suppressing region, and it always has the ability to suppress the acceleration transmission in any damping ratio situations;
3. In the suppressing region, the acceleration transmissibility decreases as the frequency ratio increases. The smaller the damping ratio, the steeper the drop.

As for the TSSS, the significant characteristics are as follows:

1. In the case of low damping ($\zeta_{1}<0.5$), there are two remarkable resonant peaks, which are located on the two sides of the undamped natural frequency $\omega_{1}$ of the inner suspension. The higher-frequency resonant peak is about 1.6 times $\omega_{1}$, and the lower-frequency one is about 0.6 times $\omega_{1}$.
2. With the increase in the damping ratio ζ, the acceleration transmissibilities of the two resonant peaks decrease, and the higher-frequency resonant peak decreases faster than the lower-frequency one;
3. The range of the suppressing region is determined by the acceleration transmissibility of the higher-frequency resonant peak, which is further decided by the damping ratio. When the damping ratio is 0, the suppressing region starts after the higher-frequency resonant peak (about $\eta_{1}>\sqrt{3}$). However, when the increased damping ratio makes the acceleration transmissibility at the higher-frequency resonant peak lower than 0 dB, a broadened suppressing region starting from about the undamped natural frequency $\omega_{1}$ (about $\eta_{1}>1$) can be achieved.
4. After the higher-frequency resonant peak, the acceleration transmissibility in the suppressing region decreases with the increase in the frequency. The smaller the damping ratio$\;\zeta_{1}$ is, the steeper the drop and the more the ability to suppress the acceleration transmissibility can be achieved.

By comparing the acceleration transmissibility spectra of two suspension systems, we can clearly observe that the TSSS has a significantly lower acceleration transmissibility than the OSSS within the high-frequency band. However, it should be discussed according to the damping ratio within the low-frequency band. If the damping ratio $\zeta_{1}>0.1$, the acceleration transmissibility at the higher-frequency resonant peak is lower than that at the same frequency for the OSSS, and we can get lower spectra within the band starting from $0.8\omega_{1}$. If the damping ratio gets lower, the acceleration transmissibility at the higher-frequency resonant peak is beyond that at the same frequency for the OSSS. In the region near the higher-frequency resonant peak, the performance of the TSSS is not so good, but the poor-performance bandwidth is not so wide.

In summary, compared with the OSSS, the TSSS has two significant advantages: first, the high-frequency acceleration transmissibility spectrum decreases more steeply with the frequency, and the acceleration transmissibility is significantly lower. Second, the low-frequency resonant frequency is lower than the resonant frequency of the OSSS, and the suppressing region can be effectively expanded by using a slightly higher damping ratio in the TSSS.

(b) The damping ratio is fixed, and the mass ratio is changed.

**Figure 8:**
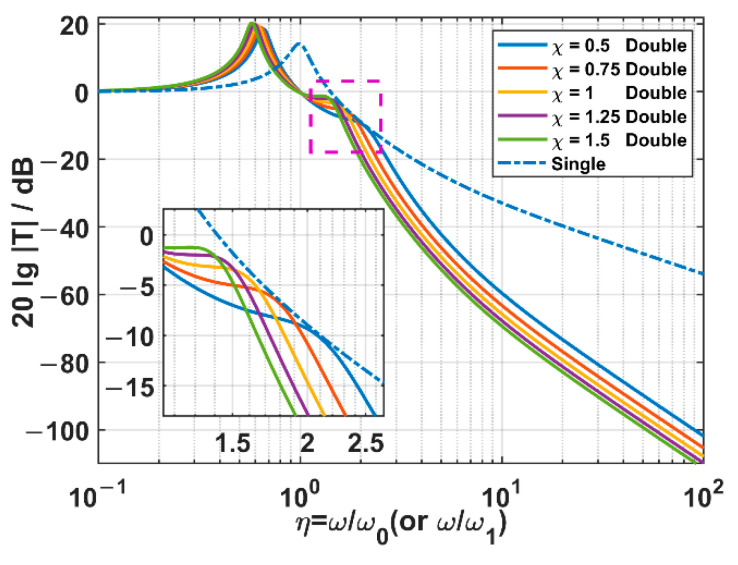
The acceleration transmissibility spectra of two suspension systems in different mass ratio cases. Among them, the dashed line is the result of the OSSS, while the solid lines are those of the TSSS.

In order to investigate the influence of the mass ratio on the acceleration transmissibility, the damping ratio is fixed as 0.1, while the mass ratio is set to be 0.5, 0.75, 1, 1.25, and 1.5, respectively. The acceleration transmissibility spectra of two suspension systems are sketched according to Equations (13) and (17), which are illustrated in Figure 8.

As for the TSSS, the acceleration transmissibility at the higher-frequency resonant peak increases with mass ratio, but the resonant frequency decreases at the same time. By comparing the spectra with that of the OSSS, we can find that the higher-frequency resonant peak almost moves along the curve of the OSSS. That is to say, the change of mass ratio does not make the acceleration transmissibility of the TSSS higher than that of the OSSS at the higher-frequency resonant frequency. Meanwhile, it is observed that the increase in the mass ratio of TSSS is conducive to the overall downward shift of spectra within the high-frequency band.

Therefore, from the perspective of reducing the high-frequency acceleration transmissibility, the enhancement of mass ratio is beneficial. However, the loading ability of the springs should also be taken into account in the practical structure when the mass of the inner frame is increased. Considering that the drop of the acceleration transmissibility spectrum is not so remarkable when the mass ratio is higher than 1.25, we take 1.25 as the optimal mass ratio.

#### 3.1.2. Comparison of the Acceleration Response of the FOVH Suspended in Two Kinds of Suspensions

(a) The mass ratio is fixed, and the damping ratio is changed.

**Figure 9:**
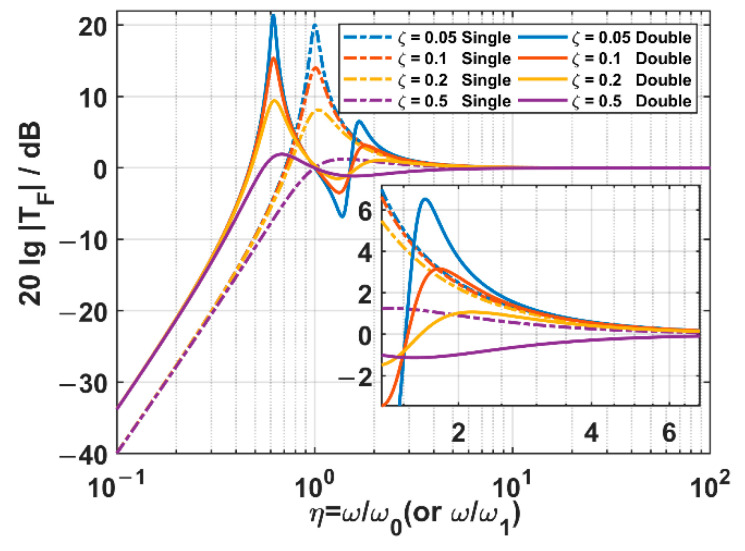
The acceleration response spectra of the FOVHs in two suspension systems in different damping ratio cases. Among them, the dashed lines are the results of the OSSS, while the solid lines are those of the TSSS.

In this part, the mass ratio is fixed to be 1, while the damping ratio is changed. Then, the acceleration response spectra of the two suspension systems are sketched in Figure 9.

It is observed that the acceleration response spectra of the FOVHs in two kinds of suspension systems are different only within the low-frequency band. As for the situation where the damping ratio is very low (ζ=0.05), the trends of the acceleration responses spectra of two suspension systems are almost the same within the band η>4. It should be noted that the FOVH in the OSSS only works well (defined as the deviation of the acceleration response being no higher than 1 dB) within the band η>4. From this point of view, the TSSS does not significantly change the working band and the in-band response of the FOVH.

(b) The damping ratio is fixed, and the mass ratio is changed.

**Figure 10:**
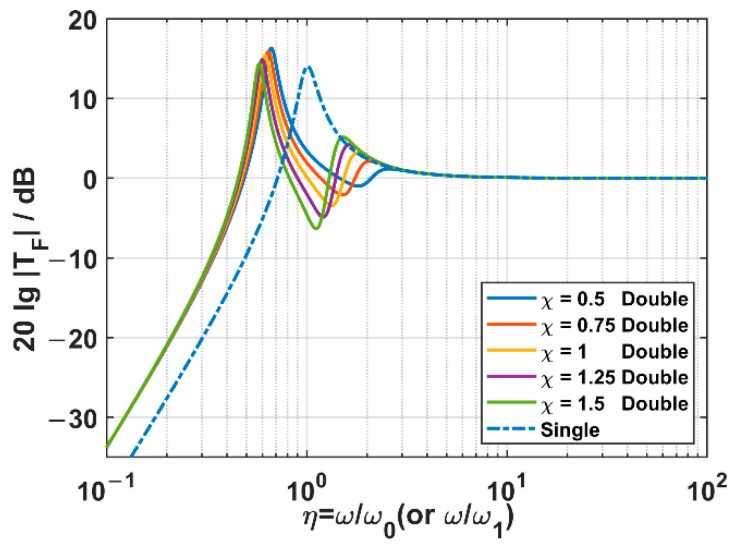
The acceleration response spectra of two suspension systems in mass ratio cases. Among them, the dashed line is the result of the OSSS, while the solid lines are those of the TSSS.

In this part, the damping ratio is fixed to be 0.1 and the mass ratio is changed. The acceleration response spectra of the FOVHs in two suspension systems are sketched in Figure 10.

The results reflect that the acceleration responses of the two suspension systems are almost the same within the band η>3. As for the optimal mass ratio of 1.25 discussed above, the spectra are almost the same when the frequency ratio is about 1.5. In this sense, compared with the OSSS, the TSSS does not significantly change the working band and the in-band response of the FOVH when the mass ratio is changed.

### 3.2. Verification of the Theory with Adams

**Figure 11:**
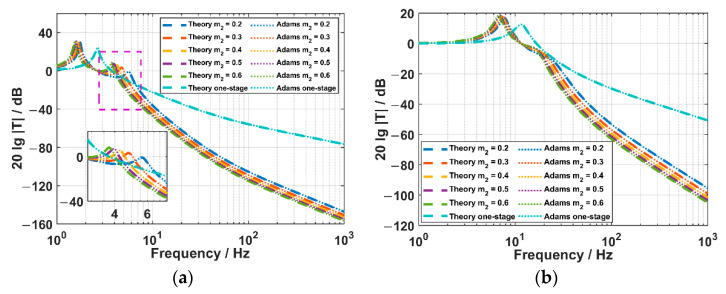

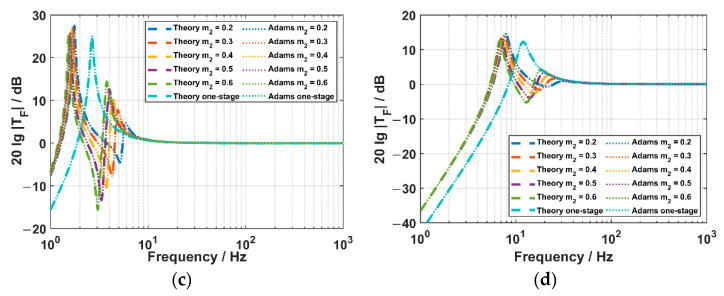
The spectra of the two suspension systems: (**a**) The X or Y acceleration transmissibility spectra of the OSSS and TSSS; (**b**) the Z acceleration transmissibility spectra of the OSSS and TSSS; (**c**) the X or Y acceleration response spectra of the FOVHs in the OSSS and TSSS; and (**d**) the Z acceleration response spectra of the FOVHs in the OSSS and TSSS.

We have also conducted the simulation with the soft Adams. In the simulation, the model is constructed with almost the same parameters of the actual structures. Among them, the masses of the outer frame and FOVH are 4.42 kg and 0.4 kg, the mass of the middle frame of the TSSS is changed from 0.2 kg to 0.6 kg, the suspending angle is 18°, the stiffnesses of the springs are all 300 N/m, while the damping coefficients are all 1 N·s/m. These parameters are also taken into Equations (8) and (10) to get the equivalent stiffnesses and damping coefficients along the principal axes, which are then used in the theory. The acceleration transmissibility spectra of the suspension systems and the acceleration response spectra of the FOVHs in the suspension systems are illustrated in Figure 11.

It is clearly observed that the Adams results show a good agreement with the theoretical ones, indicating the correctness of the equivalence method and the theory. In detail, the acceleration transmissibility spectra shown in Figure 11a reflect that the TSSS can achieve lower acceleration transmissibilities than the OSSS along X or Y direction when the frequency is higher than about 7 Hz. The frequency decreases as the mass of the middle frame increases. In particular, it turns into 4.5 Hz when $m_{2}=0.5$ kg (corresponding to the mass ratio χ=1.25). As for the acceleration transmissibility along the Z direction shown in Figure 11b, the TSSS can get lower values than the OSSS within the band higher than 10 Hz. It benefits from the suppression of the higher-frequency resonant peak as the equivalent damping coefficient is relatively higher. As for the acceleration responses shown in Figure 11c,d, the in-band acceleration responses are almost the same for the FOVHs in the OSSS and TSSS. Additionally, the working bands of the FOVHs in the OSSS and TSSS start from 9 Hz and 35 Hz along the X (or Y) and Z direction.

In conclusion, the TSSS works better in suppressing the acceleration transmission from the frame than the OSSS, and almost has no influence on the working band and in-band acceleration response of the FOVH in it.

## 4. Experiment and Discussion

In this section, the characteristics of the designed TSSS are compared with the OSSS experimentally. The actual parameters of the designed TSSS are as follows: the masses of the FOVH and the middle frame are 0.4 kg and 0.5 kg, the stiffness of each spring is 300 N/m, and the calibrated acceleration sensitivity of the FOVH is about 48 dB re rad/g [18].

**Figure 12:**
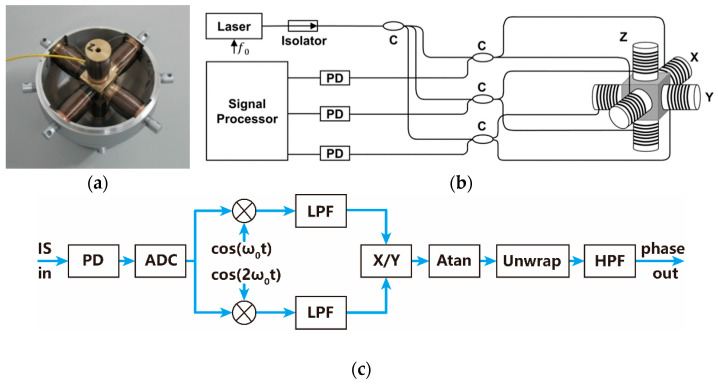
The structure diagram and signal detection flowchart of the FOVH: (**a**) The inner structure image; (**b**) the optical structure schematic diagram; and (**c**) the PGC signal detection flowchart. The laser is a semiconductor laser centered at 1550 nm; PD is the photodetector with a bandwidth of 500 kHz; C is the coupler; the Signal Processor executes the digitalization and the PGC signal detection; IS is the interference signal; ADC is the analog to digital converter; the sign ⊗ represents the multiplying; LPF is the low-pass filter; X/Y represents the division; Atan is the arc tangent process; and HPF is the high-pass filter.

Figure 12a shows the inner assembly of the FOVH, which is composed of one central mass block and six compliant cylinders with a spatially symmetrical distribution. Figure 12b illustrates the optical structure of the FOVH and its signal detection system used in the experiment. Three unbalanced Michelson interferometers are twisted around the six compliant cylinders, the arm differences of which are 0.52 m (corresponding to the optical path difference of 1.5 m). Each interferometer is wrapped on the two opposite compliant cylinders with its two sensing arms. When an ambient acceleration signal is conducted on the shell of the FOVH, it is converted into the stretching and compression of the compliant cylinders due to the inertia of the central mass block. Then, the sensing arms are elongated and shortened, leading to the phase modulation of the interference light, which is the phase signal picked by the FOVH. As shown in the figure, a peripheral signal detection system is used to detect the phase signal in the experiment. A tunable semiconductor laser centered at 1550 nm is adopted as the light source, its frequency is modulated by a sinusoidal signal with a frequency of $f_{0}$. The modulated light passes through an isolator to avoid the influence of the echo from the following devices on the laser. Afterwards, it is divided by an 1 × 3 coupler and injected into three unbalanced interferometers. Due to the arm difference of the interferometers, the frequency modulation is converted into the phase carrier, which is required by the PGC demodulation [43]. The returned interference lights are detected by three photodetectors with the same bandwidths of 500 kHz and sent into the signal processor to fulfill the phase signal detection. The flowchart of the signal detection executed in the signal processor is illustrated in Figure 12c.

In the beginning of the process, the interferometric signal is digitalized, and it is expressed by

(20) $$V=A\left\{1+v\cos\left[C\cos2\pi f_{0}t+\phi_{s}\left(t\right)+\phi_{0}\right]\right\},$$

where $A=\sigma I_{0}$ is the dc amplitude, *σ* is the photoelectric conversion efficiency, $I_{0}$ is the light power, v is the fringe visibility, $\phi_{0}$ is the initial phase that shifts slowly, and $\phi_{s}\left(t\right)$ is the phase signal picked up by the FOVH. The item $C\cos\omega_{0}t$ accounts for the generated phase carrier, where C is the phase modulation depth determined by the voltage of the frequency-modulation and $f_{0}$ is the modulation frequency. In the PGC demodulation, the interferometric signal shown in Equation (20) is firstly mixed with the fundamental harmonic and the 2nd harmonic, followed by the same low-pass filter (LPF) with the cut-off frequency lower than $f_{0}$. Then, we achieve a pair of orthogonal signals, which are the quadrature signal $q\left(t\right)=-AvJ_{1}\left(C\right)\sin\left[\phi_{s}\left(t\right)+\phi_{0}\right]$ and the in-phase signal $i\left(t\right)=-AvJ_{2}\left(C\right)\cos\left[\phi_{s}\left(t\right)+\phi_{0}\right]$. In the signals, $J_{1}\left(C\right)$, $J_{2}\left(C\right)$ are the 1st, 2nd order Bessel function of the first kind, and we have $J_{1}\left(C\right)=J_{2}\left(C\right)$ when $C=C_{0}=2.63$. After a division operation, the external phase signal $\phi_{s}\left(t\right)$ is extracted with an arc tangent operation and a high-pass filter, on the premise that the phase modulation depth C is set as 2.63 by adjusting the modulating voltage on the laser. The unwrap process is used to address the issue that the output of the arc tangent function is limited within the range from −π/2 to π/2.

### 4.1. The Characeteristics of the Acceleration Transmission of Two Suspension Systems

**Figure 13:**
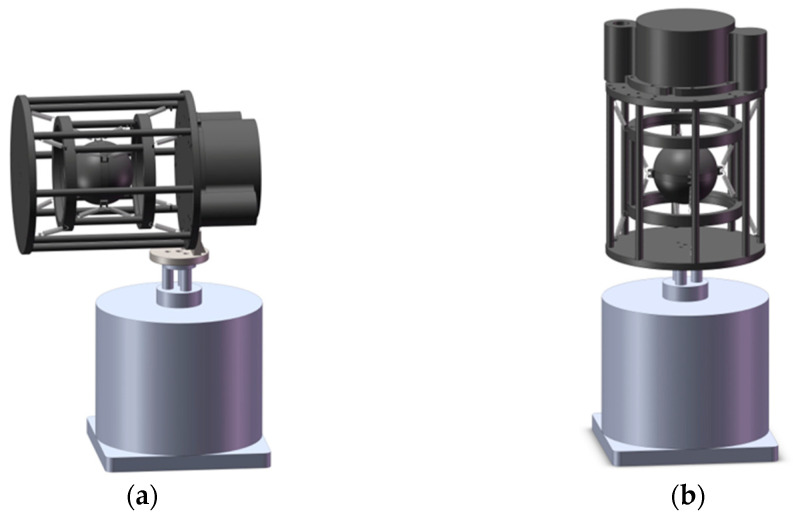
The installation diagram of the suspension systems (take TSSS for instance): (**a**) for testing the acceleration transmission along X and Y direction; (**b**) for testing the acceleration transmission along the Z direction.

In the experiment, a vibration exciter is adopted (Brüel and Kjær, type 4808), on which the suspension systems are installed with specially-designed clamps. The installation diagram is shown in Figure 13 (take the installation of the TSSS for instance). To be noted, the masses and sizes of the assemblies are specially designed to confirm that the barycenters fall within the range of the vibration cylinder for a stable measurement. In addition, a standard piezoelectric-type accelerometer is also installed on the vibration cylinder to monitor the acceleration signal sent out by the vibration exciter.

**Figure 14:**
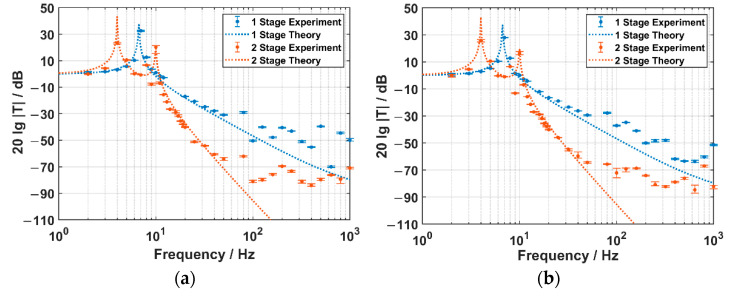

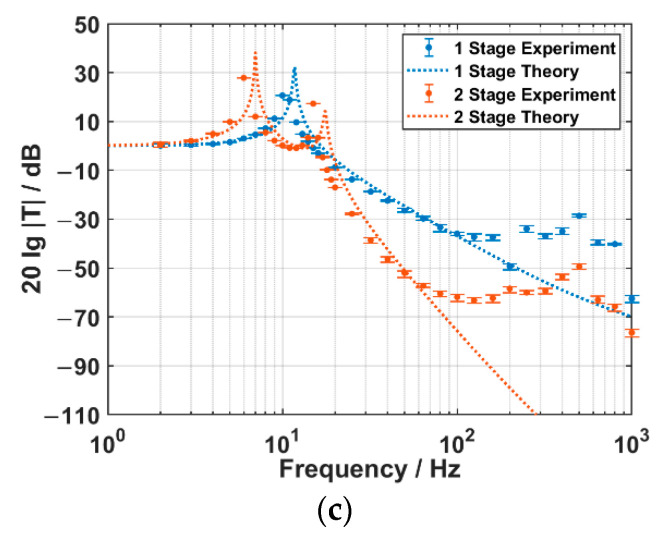
The experimental results of the acceleration transmissibility spectra for two kinds of suspension systems: (**a**) along X direction; (**b**) along Y direction; and (**c**) along Z direction.

In the experiment, the frequency of the exciting acceleration signal is swept from 2 Hz to 1 kHz. The amplitude (in dB re rad) of the phase signal picked by the FOVH is demodulated. Then, it is subtracted successively by the calibrated acceleration sensitivity (48 dB re rad/g) and the amplitude (in dB re g) of the detected signal from the standard piezoelectric-type accelerometer. Consequently, we can get the acceleration transmissibilities at various frequency points. At each frequency point, the acceleration transmissibility meaurement is repeated ten times. The experimental results are shown in Figure 14, where the upper limits of the error bars are the maximum values and the inferior ones are the minimum values of the ten-time-repeated tests. The theoretical curves are also illustrated with the dashed lines. It should be noted that the suspension angles actually deviate from 18° due to the lack of buoyancy, while the damping coefficient in air is not higher than that in water. In addition, the stiffnesses are changed due to the length change and the nonlinearity of the springs. Therefore, the simulation parameters are adjusted according to the actual state in the measurement.

It is clearly observed that the experimental results agree well with the theoretical results within the low-frequency band, while they show difference within the high-frequency band. This is because the theory is established with the inner vibration of the FOVH neglected. Actually, the experimental results are influenced by the inner vibration, especially within the high-frequency band that close to the intrinsic resonant frequency of the FOVH.

Put the high-frequency difference aside, the results show some common characteristics: Firstly, there are two chief resonant peaks for the TSSS, which locate on the two sides of the OSSS resonant peak; Secondly, the spectra of the TSSS drop steeper than those of the OSSS after the higher-frequency resonant peak. Thirdly, the TSSS has a lower acceleration transmissibility as a whole; Forthly, the acceleration transmissibilities along X and Y direction are lower than that along Z direction as a whole. These characteristics are consistent with the theoretical analysis. The experimental peaks near 500 Hz, which can be observed in both the OSSS and TSSS curves, may be caused by the structure-related resonances that originate from the installation.

The values of the acceleration transmissibilities at some typical frequency points are listed in Table 1. The results depict that, for the acceleration transmissibilities of the TSSS along X and Y direction, they are as low as −40 dB @ 20 Hz, −64 dB @ 50 Hz, and lower than −70 dB @ >100 Hz, which are 23 dB @ 20 Hz and 30 dB @ >50 Hz lower than those of the OSSS. Along the Z direction, the acceleration transmissibilities are −17 dB @ 20Hz, −52 dB @ 50 Hz, −60 @ >100 Hz, which are 8 dB @20 Hz and 25 dB @ >50 Hz lower than those of the OSSS.

In conclusion, the TSSS shows great advantage over the OSSS as far as the acceleration transmissibility is concerned.

### 4.2. The Acceleration Responses of the FOVHs in Two Suspension Systems

In this part, we use the standing-wave tude to test the acceleration sensitivities of the FOVHs in two kinds of suspension systems [37,38], which represent the acceleration responses of the FOVHs. The standing-wave tude is a tank with a rigid shell. A sound source is installed at the bottom of it, which is used to excite a stable standing-wave sound field in the tank full of water. When a standard piezoelectric-type hydrophone is placed at the same depth as the FOVH, the acceleration sensitivity of the FOVH can be acquired by [44]

(21) $$M_{A}=M_{0}+20\mathrm{lg}\left(\frac{\Delta \phi }{U_{0}}\tan kd_{0}\right)-20\mathrm{lg}\left(\frac{\Omega }{\rho c_{0}}\right),\;$$

where $M_{0}$ is the sound pressure sensitivity of the standard piezoelectric-type hydrophone in dB, Δϕ the amplitude of the phase signal received by the FOVH, $U_{0}$ is the amplitude of the voltage signal from the standard piezoelectric-type hydrophone, *k* is the wave number, $d_{0}$ is the unified depth of the two hydrophones, Ω is the circular frequency of the sound, ρ is the density of the water, and $c_{0}$ is the sound velocity in water.

**Figure 15:**
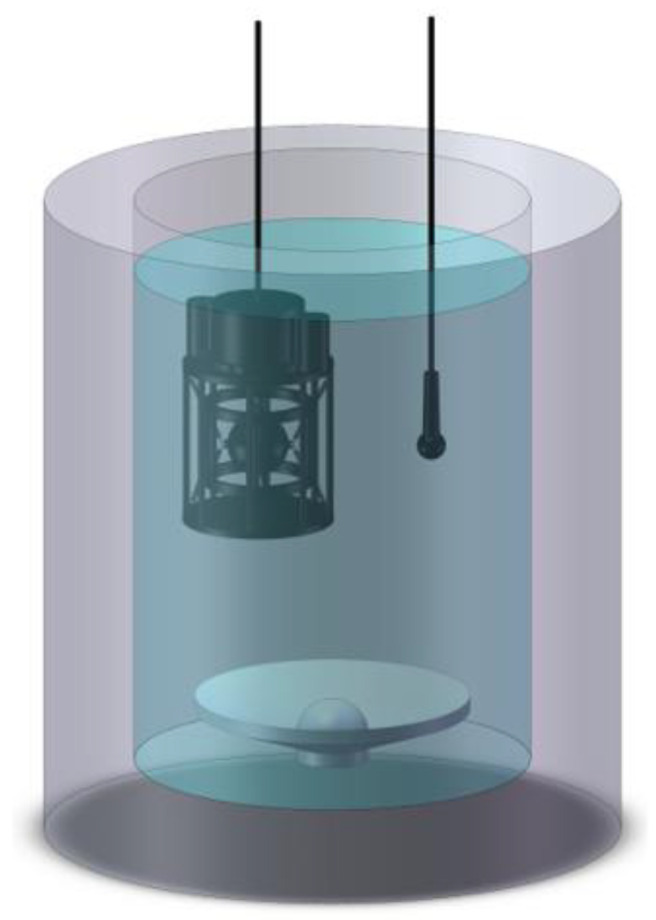
The experimental setup for testing the acceleration responses of the FOVH in the two suspension systems.

**Figure 16:**
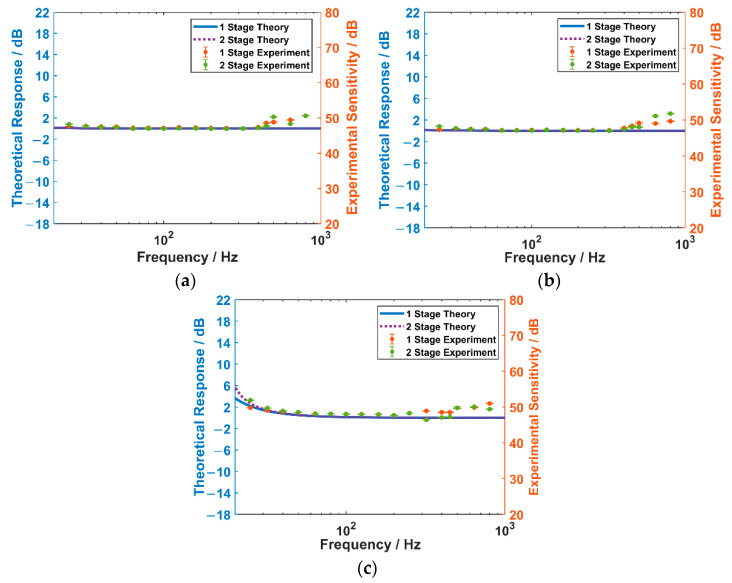
The experimental results of the acceleration sensitivities for the FOVH in two suspension systems: (**a**) X direction; (**b**) Y direction; and (**c**) Z direction.

According to Equation (21), we put the FOVH and standard hydrophone (with the sensitivity of −190.8 dB re V/μPa) at the same depth $d_{0}=0.125\;m$, which is shown in Figure 15. In order to rule out the interference of the inconsistent performances of different FOVHs, we use the same FOVH to test two suspension systems. The standing-wave sound fields with different frequencies are excited in the tube using the bottom-mounted source. Then the phase signal of the FVOH is demoduted with the optical system shown in Figure 12 and the acceleration sensitivities can be acquired afterwards. In the experiment, the acceleration sensitivity test is repeated ten times at each frequency point. The acquired acceleration sensitivities of three principal axes are shown in Figure 16, where the upper limits of the error bars are the maximum values and the inferior ones are the minimum values of the ten-time-repeated tests. The theoretical curves of the acceleration responses are also displayed.

It is shown that the experimetal results agree well with the theoretical ones within the low-frequency band, while they show some difference within the high-frequency band. The difference can also be ascribed to the neglected inner vibration of the FOVH.

By comparing the results of the OSSS and the TSSS, we can observe that the acceleration sensitivity spectra are almost all flat along the three principal axes, and the acceleration sensitivities of the FOVH in two suspension systems are almost the same within the measuring band. The little fluctuation of the FOVH in the TSSS within the high-frequency band may result from the sound field distortion caused by the large area of the bottom and top plates. Therefore, compared with the OSSS, the TSSS have limited impacts on the accerelation response of the FOVH in it.

### 4.3. The Phase Noise of the FOVH in Two Suspension Systems

In this part, a simple but intuitive experiment is designed to show the ability of the TSSS to suppress the vibration noise from the outer frame. The experiment is carried out in a quiet environment to reduce the interference of the sound transmitted via the air.

**Figure 17:**
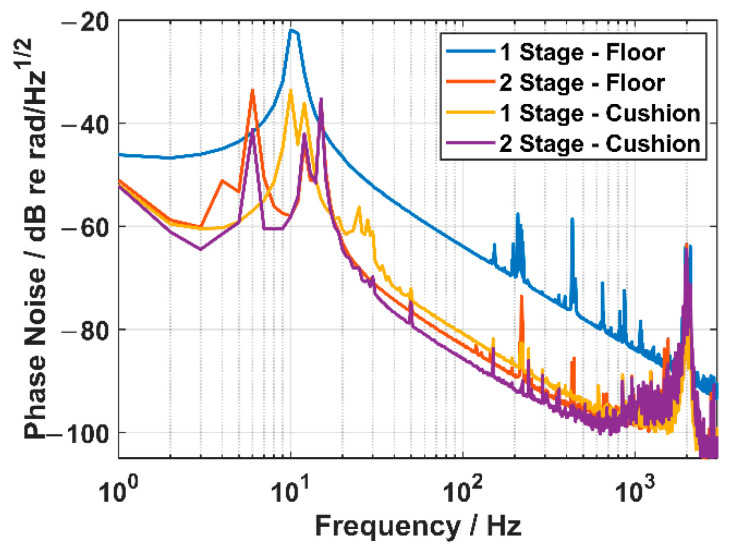
The phase noise of the Z channel of the FOVH in different suspension systems.

Firstly, the OSSS and TSSS are directly placed on the floor of the laboratory, which is located on the sixth floor of a building. The principal Z direction is vertical to the floor. In this situation, the vibration noise from the devices in the building can act on the outer frame to a great extent. Then, we test the phase noise levels of the Z channel of the FOVH in different suspension systems, and the results are shown by the blue and red lines in Figure 17. A striking contrast is observed that the phase noise of the FOVH in the OSSS is significantly higher than that of the FOVH in the TSSS, and the difference is higher than 20 dB.

Secondly, we place a sponge cushion between the suspension system and the floor to suppress the transmission of the vibration. Then, the phase noise levels of the Z channel of the FOVH in different suspension systems are also tested, which are shown by the yellow and violet lines in Figure 17. We can find that the phase noise level of the FOVH in the OSSS is suppressed greatly, which is close to that of the FOVH in the TSSS. However, the drop in the phase noise of the FOVH in the TSSS is not so remarkable.

The phenomenon indicates that it is the vibration noise from the frame that is suppressed by the TSSS. Meanwhile, the vibration noise suppression rate of the TSSS along the Z direction is over 20 dB higher than that of the OSSS. It should be noted that the phase noise levels of the X and Y channels of the FOVH are not measured because it is hard to make X or Y direction vertical to the floor, which is the optimal attitude to pick the vibration noise from the floor. In addition, the huge peak near the frequency of about 2 kHz is the resonant peak of the FOVH itself.

In order to clarify the symbols used in the paper, we have listed the symbols in Appendix A.

## 5. Conclusions

In the paper, the TSSS is designed for an interferometric acceleration-type FOVH to enhance the ability of isolating the vibration from the mooring rope. The acceleration transmissibility of the TSSS is studied theoretically and experimentally. The results show that the TSSS performs significantly better than the traditional OSSS in the vibration isolation. Typically, the vibration isolation of the TSSS is over 25 dB higher than that of the OSSS at 100 Hz. Meanwhile, it is demonstrated that the proposed TSSS has little negative influence on the acceleration response of the FOVH. Therefore, the TSSS for FOVH has the potential prospect in suppressing the mechanical noise from the mooring rope, which is beneficial to enhancing the ability of weak signal detection.

## Figures and Tables

**Table 1:** The values of the acceleration transmissibility at some typical frequencies (in dB).

Suspension System	Direction	20 Hz	50 Hz	100 Hz	250 Hz
One-stage	X	−16.93	−30.91	−50.48	−43.12
	Y	−16.52	−29.36	−37.11	−48.22
	Z	−8.77	−26.45	−35.95	−33.89
Two-stage	X	−40.17	−64.27	−81.10	−73.29
	Y	−40.10	−64.39	−72.11	−80.82
	Z	−16.99	−52.00	−61.85	−60.01
Δ	X	−23.24	−33.36	−30.62	−30.17
	Y	−23.58	−35.06	−35.00	−32.60
	Z	−8.22	−25.55	−25.90	−26.12

## Data Availability

Not applicable.

## Appendix A

In order to clarify the symbols and their units used in the paper, we list them in Table A1.

**Table A1:** The symbols used in the paper.

Symbol	Meaning	Unit
T	Acceleration transmissibility	1
$a_{A}$	Acceleration achieved by the FOVH	m/s^2^
$a_{F}$	Acceleration on the outer frame	m/s^2^
$T_{F}$	Acceleration response of the FOVH	1
m	Mass of the FOVH	kg
$F_{0}$	Amplitude of the ambient exciting force	N
$a_{P}$	Acceleration picked by the FOVH	m/s^2^
k	Stiffness coefficient of the spring	N/m
$k_{eq}$	Equivalent stiffness coefficient	N/m
δ	Small displacement along the force	m
c	Damping coefficient	N·s/m
θ	Suspending angle	°
α	Polar angle of the ambient force	°
*φ*	Azimuth angle of the ambient force	°
x	Displacement of the FOVH in the OSSS	m
X	Complex amplitude of *x*	m
*ω*	Circular frequency of the ambient excitation	Hz
u	Displacement of the outer frame	m
U	Complex amplitude of *u*	m
ζ	Damping ratio in the OSSS	1
η	Frequency ratio in the OSSS	1
$\omega_{0}$	Undamped natural frequency in the OSSS	Hz
$k_{1}$	Equivalent stiffness coefficient of the inner suspension	N/m
$c_{1}$	Equivalent damping coefficient of the inner suspension	N·s/m
$k_{2}$	Equivalent stiffness coefficient of the outer suspension	N/m
$c_{2}$	Equivalent damping coefficient of the outer suspension	N·s/m
$x_{1}$	Displacement of the FOVH in the TSSS	x
$m_{2}$	Mass of the middle frame	kg
$x_{2}$	Displacement of the middle frame	m
$\zeta_{1}$	Damping ratio of the inner suspension	1
$\eta_{1}$	Frequency ratio of the inner suspension	1
$\omega_{1}$	Undamped natural frequency of the inner suspension	Hz
χ	Mass ratio of the middle frame to the FOVH	1
A	Dc amplitude of the digitalized interference signal	V
σ	Photoelectric conversion efficiency	V/W
$I_{0}$	Light power	W
v	Fringe visibility	1
$\phi_{0}$	Initial phase	rad
$\phi_{s}\left(t\right)$	Phase signal	rad
C	Depth of the phase carrier	rad
$f_{0}$	Frequency of the phase carrier	Hz
q(t)	Quadrature signal in the PGC method	V
i(t)	In-phase signal in the PGC method	V
$M_{A}$	Acceleration sensitivity of the FOVH	dB re rad/g
$M_{0}$	Sound pressure sensitivity of the standard piezoelectric-type hydrophone	dB re V/μPa
Δ*ϕ*	Amplitude of the phase signal received by the FOVH	rad
$U_{0}$	Amplitude of the voltage signal from the piezoelectric-type hydrophone	V
k	Wave number	1/m
$d_{0}$	Unified depth	m
Ω	Circular frequency of the sound	Hz
ρ	Density of the water	kg/m^3^
$c_{0}$	Sound velocity in water	m/s
